# Embracing Progress: Thoughts on Open Access Publishing, the *JACMP*, and its $500 Article Publication Fee

**DOI:** 10.1120/jacmp.v17i5.6630

**Published:** 2016-09-08

My friend and colleague Ann Wright passed away earlier this year; you can find her memorial in the pages of *Medical Physics*. I mention her because the *JACMP* might not have come into existence without her observation that there was an urgent need for a clinical medical physics journal. I took this thought to Alex Turner, Chair of the ACMP, with the additional idea that with the emergence of the Internet, we would not need to print and mail the journal and could keep the costs down. (Alex also passed away this year; please find his memorial in the pages of the *JACMP*.) At least initially, I thought we could make this new journal:

- free to submit and review any article
- free to publish any article
- free to access any of our publications on the Internet

I thought we could sell enough banner advertising to pay for copyediting, layout editing, and the publication platform, along with a stipend for the Editor. While we did meet our objectives above, things did not work out as planned. We did not meet our initial goals for selling enough banner ads and, as a result, in 2004 we changed publishers and eliminated compensation for the Editor in order to reduce costs. In the larger arena of academic publishing, the original *JACMP* model is no longer considered possible, largely because banner ads alone cannot generate enough to keep a journal in the black for a long period of time, especially when it grows at 20% per year every year (see Table 1). Nevertheless, we were able to keep the *JACMP* publication model free, as described above, for far longer than many thought we would.

In the process, the *JACMP* entered into the Open Access Publication revolution. So much has been written about the broader arguments for open access publishing, I will not recapitulate these arguments here. Nor will I engage in the discussion over Gold versus Green open access. Consider these resources if you are interested:

http://www.nature.com/nature/focus/accessdebate/34.html

http://www.ncbi.nlm.nih.gov/pmc/articles/PMC1525322/

http://iosrjournals.org/iosr‐jhss/papers/Vol8‐issue3/B0820709.pdf

https://discussingoa.wordpress.com/

Instead, I want to focus on why in 1998 it seemed reasonable to offer clinical articles electronically and as soon as possible:

- The moral imperative � If patient lives and quality of life are at stake, it is deleterious to erect unnecessary barriers to information that could benefit them. If a medical physicist spends hundreds of hours solving a clinical problem and can publish its solution, it is ethically questionable to restrict access to that information, as it benefits both patients and other medical physicists.
- Efficiency � By processing this information electronically (remember snail mail was used back then) and eschewing print, the information could be delivered weeks earlier.
- Technology � With a flood of new technologies on our doorstep (IMRT, IGRT, SBRT, 3D and 4D imaging technologies, etc.), not to have an efficient communication venue would mean medical physicists everywhere would waste vast time and resources solving the same problems, and consequently severely limiting our rate of clinical progress and potentially the quality of our services.

So, apart from the general arguments for scholarly open access publication, clinical medical physicists have specific, urgent, and ethical needs to publish their articles with as few barriers as possible. Since assuming ownership of the *JACMP* in 2012, the AAPM, to its credit, has come to understand the open access publication model and recognize its intrinsic benefits.

**Table 1:** *JACMP* growth 2000�2015.

*Year*	# *Articles Peer‐Reviewed*	# *Articles Published*	# *Pages*	*Annual Growth Articles*	*Annual Growth Pages*
2000	unknown	19	164		
2001	unknown	29	232	53%	41%
2002	unknown	36	327	24%	41%
2003	unknown	45	385	25%	18%
2004	unknown	32	379	−29%	−2%
2005	unknown	44	492	38%	30%
2006	unknown	40	435	−9%	−12%
2007	83	48	592	20%	36%
2008	117	62	609	29%	3%
2009	164	80	817	29%	34%
2010	192	100	1098	25%	34%
2011	208	103	1229	3%	12%
2012	288	140	1484	36%	21%
2013	323	166	1805	19%	22%
2014	407	184	2199	11%	22%
2015	512	218	2613	18%	19%
**Totals**	–	1,346	14,860	–	–
Average annual growth over 15 years				**19**%	**21**%

Faced with the problem of addressing the widening deficit of the current *JACMP* business model, the following motion was initiated by the AAPM�s Strategic Planning Committee and approved by the AAPM Board of Directors at the 2015 RSNA Board Meeting:

�Recommend to the Board that the Journals Business Management Committee (JsBMC) be charged with setting a publication fee that will enable *JACMP* to at least break even by the end of 2018 while maintaining a gold open access model.�

The JsBMC agreed that the *JACMP* cannot be allowed to continue to grow at a 20% annual rate while also maintaining a deficit to the AAPM that also grows at a 20% annual rate. There was no alternative but to implement an Article Publication Charge (APC). The question became what should that charge be?

The APC is designed to recover the costs of copyediting, layout editing, proofreading, quality assurance, and electronic publication (and the cost of the platform that supports these activities). In other words, the APC pays the cost to transform an accepted article into a publication‐worthy document. The APC is not designed to recover the costs for the work of the Editor, the manuscript manager, the submission process, or the submission/peer‐review platform.

Also, what special benefit does the *JACMP* offer for which the author could reasonably be expected to pay? In paying the APC, the author retains the full copyright of the article and provides the publisher with noncommercial archival publication rights. The author may then disseminate and distribute the article to anyone without restriction. The author retains full commercial rights to the article and can negotiate to have it posted on any vendor site. Occasionally, I receive requests to reprint content from a *JACMP* published article; I refer all such requests to the author.

So to summarize, the author receives the following benefits without cost:

- the support of the peer‐review platform,
- the support of the Manuscript Manager,
- the support of the Section Editor, which is volunteer labor,
- the support of the Reviewers, which are also volunteer labor, and
- the support of the Editor during the review and editing process

And for the $500 APC, which is typically a small fraction of the costs (personal, institutional, and grant) to gather and analyze data, to write the manuscript, and to navigate the review and publication process, the author receives:

- the support of the editorial platform,
- the support of the publishing platform in perpetuity,
- the support of the copyeditor,
- the support of the layout editor,
- the distribution of the article potentially to any of the 3 billion individuals with web access, and
- the full and unrestricted copyright ownership of the article.

One of the purposes of the $500 APC is to strengthen and solidify the presence of the *JACMP* in the medical physics publication community. Consider these perspectives:

The *JACMP* is now stronger than ever as it continues to evolve as the premier clinical journal complementary to Medical Physics. As many of the world�s scientific journals are or are becoming open access, *JACMP* remains at the forefront of that movement. The $500 charge helps solidify the journal�s optimistic future. The Journal has managed to keep the publication fee very small compared with fees of other journals in our field, and this fee represents a relatively small cost compared with the effort of performing the work upon which an article is based and the publication efforts of the authors and institutions.

*Medical Physics* and *JACMP* are intended to serve as separate, complementary AAPM journals, each with a different purpose and persona in the community:

- AAPM Ownership � Both journals are valued AAPM journals in which AAPM members take pride and which advance the mission of the AAPM.
- Journal Content � Both journals will cooperate to improve, review, and update specified journal content to be inclusive of AAPM needs yet as mutually exclusive as possible. Together the two journals will meet the needs of research medical physicists, professional clinical medical physicists, medical physics educators, and medical physics students and trainees.
- Publicity � Both journals are to be distinct with a strong branding. Journal content can be publicized through publications in AAPM journals and the newsletter, presentations at chapter and national AAPM meetings, as well as by partnering with the publisher to establish the presence of both journals in the international medical physics community.

The *JACMP* is truly a worldwide journal, which publishes articles from every continent. So consider how the $500 APC compares to that of other international open access journals in the radiology, nuclear medicine, imaging, and radiation oncology space. Table 2 shows all such journals with a Thomson ISI Impact Factor, while eliminating three that have only a regionalimpact.

I realize that for some readers this explanation of why and how the APC came about might be less than persuasive. I can understand why, since for many years, the *JACMP* was free to publish. But consider this benefit was based on much volunteer effort. A typical article takes several hours of labor for the Editor to guide from submission to publication. Add time for the manuscript manager to oversee peer‐review; until recently, the Editor donated his time to perform that function. The total became several man‐years of volunteer editorial labor that built and supported the *JACMP*, not counting the contributions of the many Section Editors and Reviewers, as well. We donated this time because we wanted the *JACMP* to succeed and *JACMP* articles to be published without cost to the authors for as long as possible.

As seen in Table 2, the $500 fee is less than one‐third the cost of that of other related journals in the broad publication space of the *JACMP*. The other side of this benefit is that the *JACMP* author community represents about 3,000 AAPM members out of our membership of almost 9,000. If AAPM were to continue to support the full cost of publication in the *JACMP*, a large benefit would accrue to just one‐third of the membership, not to mention the many *JACMP* authors who are not AAPM members.

**Table 2:** Impact and cost of international open access Thomson ISI journals in the radiology, nuclear medicine, imaging, and radiation oncology publishing space.^�^

How does the $500 Article Publication Charge (APC) compare to those of other international open access journals in the radiology, nuclear medicine, imaging space? (assumes no discounts).						
*Rank*	*Full Journal Title‐Open Access*	*Journal Impact Factor (Rank)*	*Eigenfactor Score (Rank)*	*Average Rank*	*APC*	*Publisher*
1	*Biomedical Optics Express*	3.344 (2)	0.019530 (1)	1.5	$1,489.00	OSA Publishing
2	*Journal of Cardiovascular Magnetic Resonance*	5.752 (1)	0.012020 (3)	2.0	$2,145.00	Biomed Central
3	*Radiation Oncology*	2.466 (3)	0.014120 (2)	2.5	$2,145.00	Biomed Central
4 tie	*EJNMMI Research*	1.761 (4)	0.002740 (7)	5.5	$1,750.00	Springer
4 tie	*Journal of Radiation Research*	1.536 (7)	0.004470 (4)	5.5	$1,600.00	Oxford
6	*Korean Journal of Radiology*	1.592 (6)	0.003570 (6)	6.0	$1,000.00	KAMJE
**7 tie**	***Journal of Applied Clinical Medical Physics***	**1.444 (9)**	**0.003870 (5)**	**7.0**	**$500.00**	**Multimed**
7 tie	*BMC Medical Imaging*	1.663 (5)	0.001690 (9)	7.0	$2,145.00	Biomed Central
9	*Cancer Imaging*	1.470 (8)	0.001940 (8)	8.0	$2,145.00	Biomed Central

^�^ I recently learned that there is a new clinical and science medical physics journal being planned by the European Society for Radiotherapy and Oncology (ESTRO) http://www.estro.org/; it is called *Physics and Imaging in Radiation Oncology*, or phiRO http://phiro.science/. Mr. Joe Butler, a representative of the publisher Elsevier, reports the Article Publication Fee (APC) is 1494 euros, or approximately $1700. The first issue of this new journal has not yet been published, and there are no articles in press as of late August, 2016. It will be several years before an Impact Factor could be reported for this new publication.

If you would consider the unique need of the clinical medical physics community to have an open access clinical journal, I hope you see how the *JACMP* meets that need. I trust you will also conclude that publishing in the *JACMP* is a good deal and the $500 APC is a reasonable price to pay to provide the benefit of our scholarly contributions without barriers to the worldwide medical physics community.

## ACKNOWLEDGMENTS

I want to thank Timothy Solberg and Per Halvorsen, Associate Editors‐in‐Chief of the *JACMP*, for their valuable and perceptive comments. Special thanks also to Ken Hogstrom and Sam Armato for their insightful perspective and support.

## COPYRIGHT

This work is licensed under a Creative Commons Attribution 3.0 Unported License.

Michael D. Mills, PhD

Editor‐in‐Chief

